# Mapping local recurrence after radical resection of pancreatic body and tail cancer for target volume design

**DOI:** 10.3389/fonc.2026.1651747

**Published:** 2026-05-20

**Authors:** Xia Xiao, Yi-Qiu Li, Xiao-Ting Xu

**Affiliations:** Department of Radiation Oncology, The First Affiliated Hospital of Soochow University, Suzhou, Jiangsu, China

**Keywords:** carcinoma, pancreatic ductal, clinical study, clinical target volume, neoplasm recurrence, local, radiotherapy, adjuvant

## Abstract

**Background:**

To construct a three-dimensional (3-D) mapping model of local recurrence in patients with pancreatic body and tail cancer (PDTC) after radical resection, and to assess the coverage of clinical target volume (CTV) defined by Radiation Therapy Oncology Group (RTOG) consensus guidelines for PDTC on local recurrence lesions.

**Methods:**

Patients who experienced local recurrence after radical resection of PDTC between January 2013 and March 2024 were retrospectively analyzed. The geometric center of each local recurrence focus was marked as a point to determine its spatial location relative to the celiac artery (CA) or superior mesenteric artery (SMA). And a 3-D local recurrence map model was constructed based on the CT template image of a patient who underwent radical resection. Subsequently, the coverage of the postoperative CTV, as defined by RTOG consensus guidelines, on local recurrence sites was evaluated.

**Results:**

A 3-D local recurrence map with 59 local recurrence points of 41 PDTC patients was generated. The mean distance from all local recurrence points to the CA was 3.14 cm (range, 0.67 - 8.68 cm), and the mean distance to the SMA was 3.23 cm (range, 0.66 - 8.59 cm). A total of 24 recurrence points were not covered by the CTV_RTOG_. Specifically, among the 24 recurrence points closer to the CA, 15 were not covered by CTV_RTOG_. Among the 35 recurrence points closer to the SMA, 9 were not covered by CTV_RTOG._ Among patients who received chemotherapy, 12 recurrence points were not covered by CTV_RTOG_, including 9 near the CA and 3 near the SMA. Among patients who did not receive chemotherapy, 12 recurrence points were not covered by CTV_RTOG_, including 6 near the CA and 6 near the SMA. There was no statistically significant difference in the coverage of recurrence points by CTV_RTOG_ between the two groups (P = 0.400).

**Conclusions:**

We constructed a 3-D anatomical recurrence map for PDTC following radical resection and demonstrated that 40.68% of local recurrence sites fall outside the CTV_RTOG_. These findings may provide valuable insights into optimizing the design of target volumes for postoperative radiotherapy in PDTC.

## 1 Introduction

Pancreatic cancer is a highly aggressive tumor of the digestive system, ranking 11th in incidence but 3th in mortality rate (1). Although approximately 20% of pancreatic cancer patients are diagnosed at an early stage and undergo radical resection surgery, 80% experience recurrence or metastasis postoperatively, with a 5-year survival rate of only 3% (2, 3). Preoperative and postoperative adjuvant therapy can enhance the cure rate and prolong survival after pancreatic cancer surgery. The efficacy of postoperative adjuvant chemotherapy has been validated (4, 5). Several retrospective studies have explored the effectiveness of adjuvant chemoradiotherapy for pancreatic cancer, indicating that postoperative chemoradiotherapy may improve local control and overall survival (OS) in patients with positive surgical margins or lymph node involvement (6, 7). Based on our prior research, we found that postoperative adjuvant radiotherapy for tumors located in the body and tail of the pancreas was challenging to extend survival and should be applied cautiously. This might be attributed to the radiotherapy sensitivity of pancreatic body and tail cancer (PDTC) cells and the design of the radiotherapy target area (8).

Currently, the delineation of postoperative adjuvant radiotherapy target areas for PDTC primarily refers to the RTOG 0848 guidelines for pancreatic head cancer (9), with limited studies validating whether these target areas cover high-risk regions of local recurrence. Some studies have designed a new clinical target volume for postoperative pancreatic cancer by generating a local recurrence map following pancreaticoduodenectomy in pancreatic cancer patients, aiming to effectively encompass recurrence points and enhance the therapeutic gain ratio (10–12). However, data specific to PDTC remain scarce. Therefore, we constructed a local recurrence map after radical resection in resectable PDTC patients and evaluated the coverage of CTV_RTOG_ for local recurrence sites based on the RTOG pancreatic cancer consensus guidelines.

## 2 Materials and methods

### 2.1 Patient selection

Clinical, pathological, imaging, and treatment follow-up data of patients with locally recurrent primary PDTC after radical resection were retrospectively collected from the First Affiliated Hospital of Soochow University between January 2013 and March 2024. The inclusion criteria were as follows: (1) age over 18 years; (2) a history of radical resection for PDTC; (3) pathological confirmation of pancreatic ductal adenocarcinoma or its histological subtypes; (4) no prior neoadjuvant therapy and no postoperative adjuvant radiotherapy; (5) radiologically confirmed local tumor recurrence, with or without distant metastasis, based on postoperative CT, MRI, or PET-CT imaging, along with availability of high-quality CT images clearly delineating the recurrent lesion; (6) availability of complete clinicopathological data and follow-up information including survival outcomes. Exclusion criteria were defined as: (1) receipt of postoperative adjuvant radiotherapy; (2) presence of a second primary malignancy; (3) insufficient follow-up data, particularly regarding recurrence status. All patients underwent comprehensive imaging assessments at regular intervals of 2-3 months following surgery. Postoperative adjuvant chemotherapy, defined as completion of at least two cycles of a gemcitabine-based or fluorouracil-based regimen, was administered to eligible patients; individuals who did not meet this criterion were classified as having not received adjuvant chemotherapy. Local recurrence was defined as the presence of soft tissue masses or nodules in the surgical area, soft tissue encasement around peripancreatic vessels such as the superior mesenteric artery (SMA) and celiac artery (CA), and retroperitoneal lymph node metastasis, either alone or in combination with distant metastasis. This study was approved by the institutional review board (Approval No. 2025327). Informed consent was waived due to the retrospective nature of the study and the use of anonymized clinical data for analysis.

### 2.2 Local recurrence mapping

The CT image data of forty-one patients with local recurrence after pancreatic body and tail cancer surgery were imported into the Varian Eclipse 13.6 treatment planning system at the Radiation Oncology Center of the First Affiliated Hospital of Soochow University. To ensure accurate and reproducible identification and annotation of recurrent lesions, the original volumetric datasets were reconstructed on a dedicated radiological post-processing workstation using a slice thickness of 1.0 mm. These thin-section axial images constituted the foundational dataset for advanced three-dimensional visualization techniques—including multiplanar reconstruction (MPR), volume rendering (VR), and curved planar reconstruction (CPR)—specifically applied to facilitate precise, contour-based delineation of target lesion regions. The identification of local recurrence sites and the contouring of tumor recurrence foci were conducted by two chief physicians from the Department of Radiation Oncology, based on comprehensive assessments of clinical records and multimodal imaging data. Discrepancies were resolved through discussion and consensus, with final decisions on contentious cases made by the corresponding author, while the first author performed the delineation procedures. Professor Xiao-Ting Xu from the Department of Radiation Oncology at the First Affiliated Hospital of Soochow University, who possesses extensive clinical expertise in radiation oncology, provided primary guidance for the process in accordance with established guidelines, including those issued by the RTOG. A physicist then generated the geometric center points of these recurrences by adding radiation fields to the delineated regions. Based on these center points, the positions of all tumor recurrences were marked as points on the images. The vascular contours of the CA and SMA were delineated according to the region-of-interest generation steps outlined in the RTOG 0848 guideline. Specifically, the CA was delineated from its origin along the natural contour for 1 cm, while the SMA was delineated from its origin along the natural contour for 3 cm. The geometric centers of both vessels were generated by adding radiation fields to their respective delineations. Subsequently, the distances from each recurrence point to the centers of the CA and SMA were calculated for each patient, and the closer vessel (CA or SMA) to the recurrence point was identified and recorded. Additionally, the left-right body width, anterior-posterior body thickness, and height from the upper edge of T12 to the lower edge of L2 at the center points of the CA or SMA were measured on the CT images of each patient.

A CT image from a patient who underwent radical surgery for PDTC was selected as a template. On the 1mm-thick slices generated through three-dimensional reconstruction, the contours of the CA and SMA were delineated, and their center points were obtained. Based on the proportional ratios of body width, body thickness, and height between the template patient and each individual patient, the X, Y, and Z coordinates of the distances from each recurrence point to the CA or SMA were proportionally scaled. Finally, all adjusted coordinate data were marked on the CT image of the template patient, generating a three-dimensional local recurrence map comprising fifty-nine recurrence lesions.

### 2.3 Evaluation of the coverage of local recurrence sites

We evaluated the coverage of the target volume on local recurrence sites on the template CT using the RTOG consensus guidelines for postoperative radiotherapy of pancreatic head cancer, excluding hepatic portal lymph nodes. The region of interest included the postoperative tumor bed, anastomosis site, 1.0 cm proximal to the most proximal end of the CA vessel, 3.0 cm proximal to the SMA vessel, and the aorta extending from the highest level of the CA, postoperative tumor bed, or SMA to the lower edge of L3. Subsequently, the CA, SMA, anastomosis site, and postoperative tumor bed were expanded by 1.0 cm in all directions. The aorta was expanded by 3.0 cm to the right, 1.0 cm to the left, 2.5 cm anteriorly, and 0.2 cm posteriorly. CTV_RTOG_ was created by combining the above regions of interest through Boolean logic operations. A radiation oncologist delineated the postoperative radiotherapy target volume on the template CT.

### 2.4 Statistical analysis

All variables were categorical, and descriptive statistics were summarized as frequencies (n) and percentages (%). Patients were stratified into two groups—adjuvant chemotherapy versus no adjuvant chemotherapy—based on receipt of postoperative systemic treatment. OS and disease-free survival (DFS) were estimated using the Kaplan-Meier method, and corresponding survival curves were generated. OS was defined as the time interval from the date of radical resection to death from any cause. DFS was defined as the time from radical resection to the earliest occurrence of tumor recurrence, distant metastasis, or death. Censoring occurred at last follow-up or study closure for patients who remained alive and free of events. Between-group differences in OS and DFS were assessed using the Log-rank test. The distribution of postoperative recurrence sites across groups was compared using either the chi-square test or Fisher’s exact test, selected according to the minimum expected cell count (chi-square if all expected counts ≥5; otherwise Fisher’s exact test). All hypothesis tests were two-sided, with statistical significance defined as P < 0.05. Statistical analyses were performed using SPSS version 25.0.

## 3 Results

### 3.1 Characteristics and recurrence patterns of patients

The basic clinical, pathological, and treatment information of the patients is summarized in **Table 1**.  A total of forty-one patients were included in this study, comprising twenty males and twenty-one females. Among them, twenty-eight patients were aged over sixty years at the time of surgery. Of these, forty underwent radical resection for PDTC, while one underwent total pancreatectomy. Laparoscopic surgery was performed in eleven cases, vascular reconstruction in five cases, and lymph node dissection involving ≥12 nodes in eight cases. Histopathological findings revealed forty-one cases of ductal adenocarcinoma and its subtypes, including twenty-six cases with poor-to-moderate differentiation, eight cases with vascular invasion, two cases with positive resection margins, eighteen cases with nerve invasion, and four cases with cancer nodules. As the present study forms part of this ongoing research series, the consistent application of the previously established 3.5 cm cutoff-corresponding to the median value-was maintained to ensure methodological continuity and comparability across analyses. Tumor size was > 3.5 cm in twenty-eight cases, with staging as follows: T_1_ (n=4), T_2_ (n=19), T_3_ (n=18); N_0_ (n=19), N_1_ (n=18), N_2_ (n=4). Preoperative CA199 was elevated in twenty-three cases, preoperative CEA in eight cases, preoperative CA125 in six cases, and postoperative CA199 in twelve cases. Among all patients, eighteen received adjuvant chemotherapy (fluoropyrimidine-based or gemcitabine-based regimens), while twenty-three did not receive adjuvant treatment.

**Table 1:** Basic characteristics of 41 patients with pancreatic body and tail cancer.

Characteristics	N (%)
Gender	
Male	20 (48.78)
Female	21 (51.22)
Age, y	
≤ 60	13 (31.71)
> 60	28 (68.29)
Year of surgery	
2013-2018	15 (36.59)
2019-2024	26 (63.41)
Surgery	
Laparoscopic	11 (26.83)
Open	30 (73.17)
Vascular reconstruction	
Yes	5 (12.20)
No	36 (87.80)
Surgical approach	
Radical resection of pancreatic body and tail cancer	40 (97.56)
Total pancreatectomy	1 (2.44)
Number of lymph node dissections	
< 12	33 (80.49)
≥ 12	8 (19.51)
Degree of differentiation	
Poor-to-moderate	26 (63.41)
Moderate-to-well	14 (34.15)
Unknown	1 (2.44)
Pathological type	
Ductal adenocarcinoma	40 (97.56)
Other	1 (2.44)
Vascularization	
Positive	8 (19.51)
Negative	33 (80.49)
Margin	
Positive	2 (4.88)
Negative	39 (95.12)
Carcinoma nodules	
Existence	4 (9.76)
Non-existence	37 (90.24)
Nerve invasion	
Yes	18 (43.90)
No	23 (56.10)
Tumor diameter, cm	
≤ 3.5	13 (31.71)
> 3.5	28 (68.29)
T stage	
T_1_	4 (9.76)
T_2_	19 (46.34)
T_3_	18 (43.90)
N stage	
N_0_	19 (46.34)
N_1_	18 (43.90)
N_2_	4 (9.76)
Preoperative CA199 level, U/ml	
≤ 37	9 (21.95)
> 37	23 (56.10)
Unknown	9 (21.95)
Preoperative CEA level, µg/L	
≤ 5	23 (56.10)
> 5	8 (19.51)
Unknown	10 (24.39)
Preoperative CA125 level, U/ml	
≤ 35	24 (58.54)
> 35	6 (14.63)
Unknown	11 (26.83)
Postoperative CA199 level, U/ml	
≤ 37	13 (31.71)
> 37	12 (29.27)
Unknown	16 (39.02)
Postoperative chemotherapy	
Yes	18 (43.90)
No/unknown	23 (56.10)

%: The proportion of cases attributable to each research factor relative to the total number of observed cases.

Among all patients, twenty-two cases (53.66%) experienced local recurrence only, while nineteen cases (46.34%) had distant metastasis, including liver metastasis (n=14, 34.15%), lung metastasis (n=5, 12.20%), peritoneal metastasis (n=5, 12.20%), and bone metastasis (n=2, 4.88%). Specific details are provided in **Table 2**.

**Table 2:** Recurrence patterns of pancreatic body and tail cancer.

Type of recurrence	N (%)
Local only	22 (53.66)
Local and distant metastasis	19 (46.34)
Combined metastasis	
Liver	14 (34.15)
Lung	5 (12.20)
Peritoneal	5 (12.20)
Bone	2 (4.88)

%: The proportion of cases attributable to each research factor relative to the total number of observed cases.

**Figure 1:**
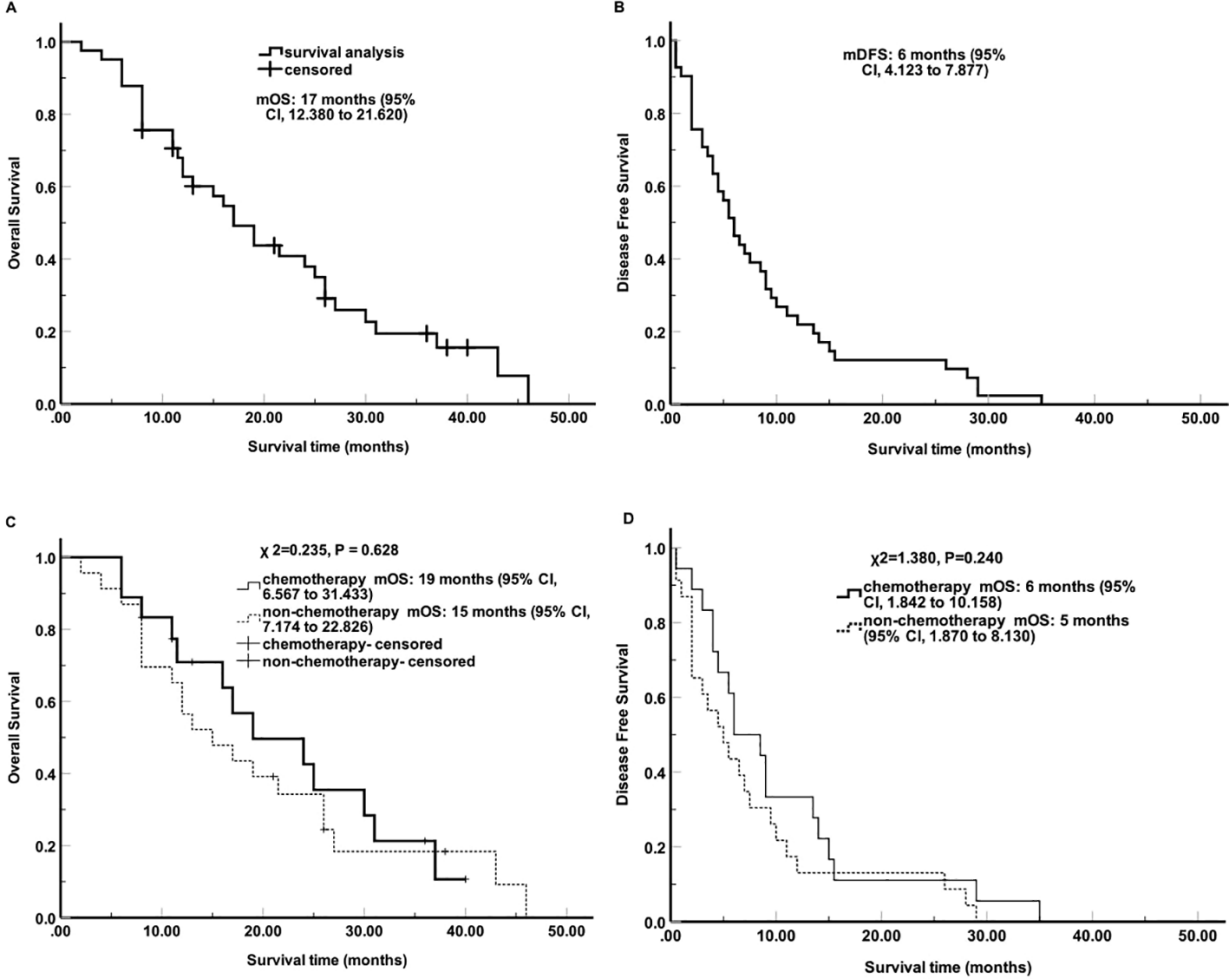
Digitally reconstructed radiograph of local recurrences. **(A)** Anteroposterior and **(B)** lateral views of local recurrence plots in relation to the celiac artery (yellow), and superior mesenteric artery (purple) after radical resection for patients undergoing adjuvant chemotherapy (blue) and no adjuvant chemotherapy (red). **(C)** anteroposterior and **(D)** lateral views of local recurrence plots closing to the celiac artery (orange), and superior mesenteric artery (green).

As shown in **Figure 1**, the median time to local recurrence after radical resection for PDTC was 6 months (95% CI, 4.123 - 7.877), with a median overall survival of 17 months (95% CI, 12.380 - 21.620). The research report further incorporated Kaplan-Meier curve analysis to assess the impact of postoperative adjuvant chemotherapy on local recurrence. The results demonstrated that patients who did not receive adjuvant chemotherapy were more likely to experience higher rates of local recurrence and had shorter survival times compared to those who underwent chemotherapy, although these differences did not reach statistical significance (P > 0.05).

### 3.2 Three-dimensional local recurrence map

**Figure 2:**
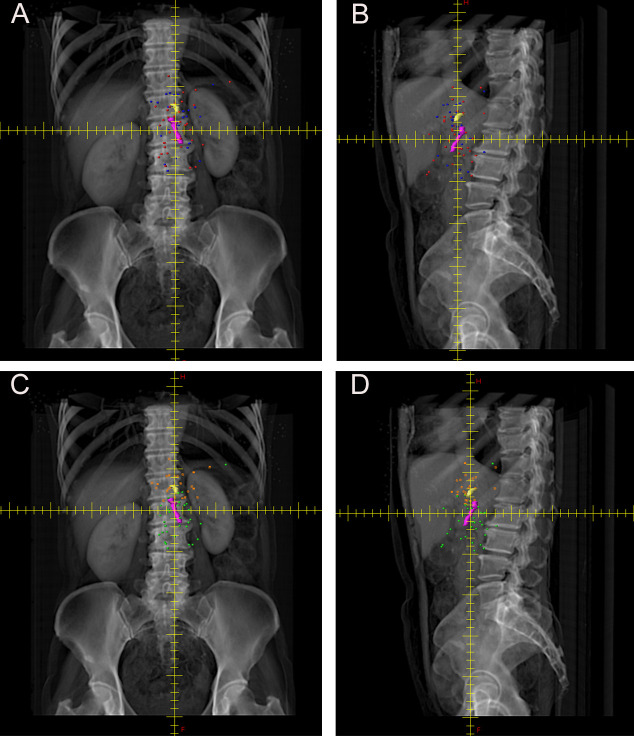
Coverage of local recurrence sites. **(A)** Anteroposterior and **(B)** lateral views of local recurrence plots after radical resection for patients undergoing adjuvant chemotherapy (blue) and no adjuvant chemotherapy (red) within CTVRTOG (yellow). **(C)** Anteroposterior and **(D)** lateral views of local recurrence plots closing to the celiac artery (orange), and superior mesenteric artery (green) within CTVRTOG (yellow).

Postoperative CT confirmed fifty-nine local recurrence lesions in these forty-one patients. All recurrence points were marked on the template patient’s CT image, generating a three-dimensional (3-D) local recurrence map comprising fifty-nine recurrence lesions. The 3-D map of local recurrence after PDTC surgery is presented in **Figure 2**. It could be observed that most local recurrence sites were distributed around the coordinate vessels, CA and SMA. The distances from all recurrence points to CA and SMA were 3.14 cm (range, 0.67 - 8.68 cm) and 3.23 cm (range, 0.66 - 8.59 cm), respectively. Among them, orange and green dots on the template CT indicated recurrence points closer to CA and SMA, respectively. There were twenty-four recurrence lesions closer to CA, with a median distance of 2.07 cm (range, 0.67 - 6.79 cm), and thirty-five recurrence lesions closer to SMA, with a median distance of 2.56 cm (range, 0.66 - 8.57 cm). Blue and red dots were used to mark the recurrence points of patients who received chemotherapy and those who did not, respectively.

Among the twenty-four recurrence points in patients who received chemotherapy, thirteen were closer to CA and eleven were closer to SMA. Among the thirty-five recurrence points in patients who did not receive chemotherapy, eleven were closer to CA and twenty-four were closer to SMA. No statistically significant difference was observed between the two groups (P = 0.081), as detailed in **Table 3**.

**Table 3:** The anatomic distribution of fifty-nine sites of local recurrence in forty-one patients.

Distribution of local recurrence	All (n=59)	Adjuvant chemotherapy (n=24)	Non-adjuvant chemotherapy(n=35)	P value
Celiac artery	24	13	11	0.081
Superior mesenteric artery	35	11	24	

### 3.3 Evaluation of target volume coverage

**Figure 3:**
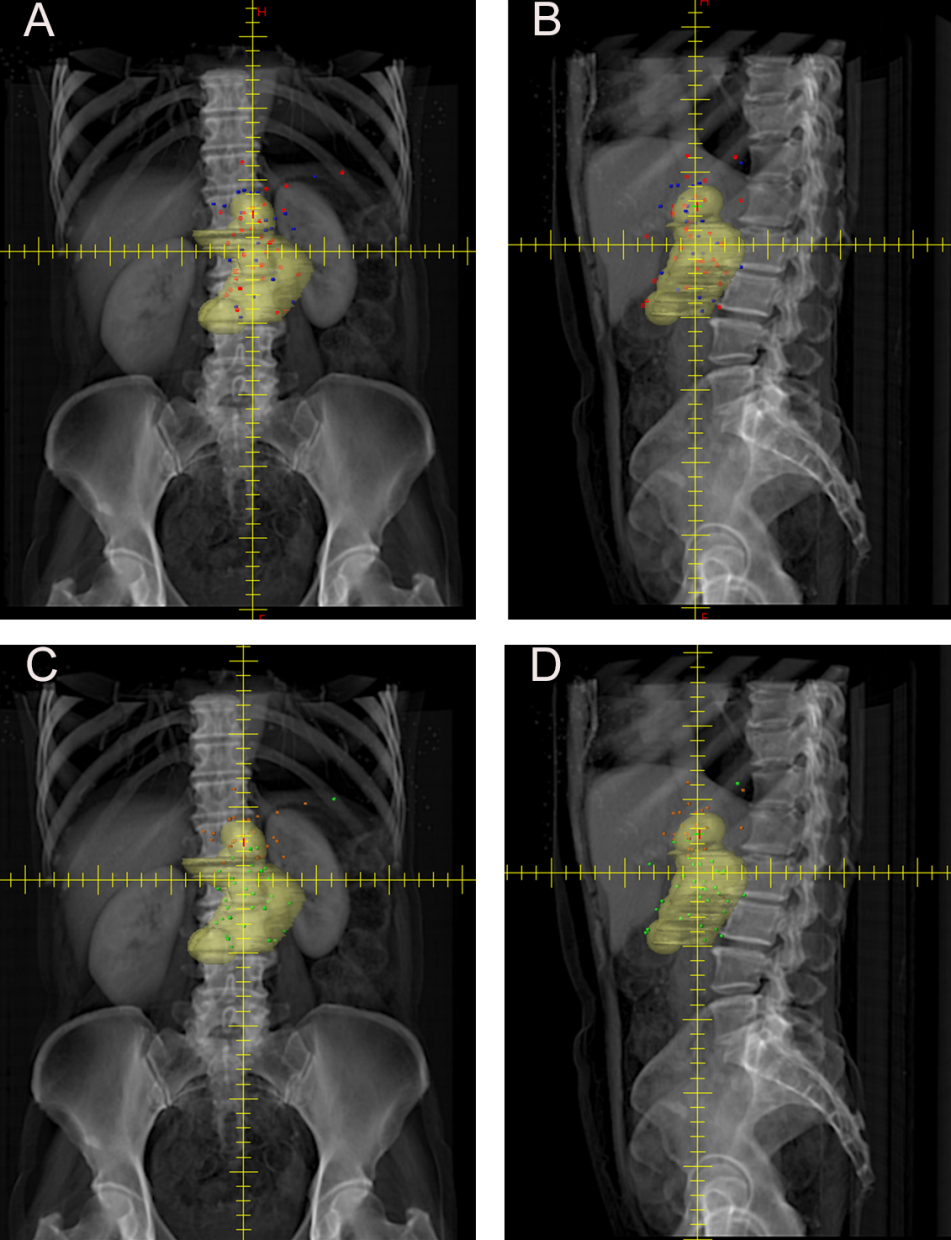
Kaplan-Meier curves for OS and DFS. **(A)** OS of all patients; **(B)** DFS of all patients; **(C)** OS for patients who received chemotherapy and those who did not receive chemotherapy; **(D)** DFS for patients who received chemotherapy and those who did not receive chemotherapy. OS, overall survival; DFS, disease-free survival.

When CTV_RTOG_ was overlaid on the recurrence sites, twenty-four of the fifty-nine local recurrence sites (40.68%) were not covered by CTV_RTOG_, as shown in **Figure 3**.

Among the uncovered recurrence points, fifteen were near CA and nine were near SMA. Among the twelve recurrence points in patients who received chemotherapy, nine were near CA and three were near SMA. Among the twelve recurrence points in patients who did not receive chemotherapy, six were near CA and six were near SMA. No statistically significant difference was observed between the two groups (p = 0.400), as detailed in **Table 4**.

**Table 4:** The anatomic distribution of local recurrence outside CTV_RTOG._.

Distribution of local recurrence	All (n=24)	Adjuvant chemotherapy (n=12)	Non-adjuvant chemotherapy (n=12)	P value
Celiac artery	15	9	6	0.400
Superior mesenteric artery	9	3	6	

## 4 Discussion

In this study, we investigated the anatomical distribution of local recurrence following radical resection of PDTC and generated a detailed anatomical map of recurrence relative to the CA and SMA. Previous studies have described recurrence distributions using recurrence maps (10–13). A 2013 study generated local recurrence maps following pancreaticoduodenectomy in patients with resectable pancreatic ductal adenocarcinoma. However, the inclusion of patients who received adjuvant radiotherapy may have influenced the results, and the study primarily focused on patients with pancreatic head cancer, lacking data on the body and tail of the pancreas (10). A 2016 study excluded patients who received adjuvant radiotherapy due to the small number of pancreatic body and tail cancer patients (11). A 2019 study found that most postoperative local recurrences in patients with pancreatic head and body cancer occurred in a small area around the CA and SMA. However, it did not include data on the tail of the pancreas (12). Notably, building on these findings, this study reconstructed the recurrence patterns of PDTC after surgery, innovatively accounting for variations in body width, thickness, and height to reproduce recurrence locations. Additionally, geometric center points were established by defining physical radiation fields, enabling a more accurate representation of recurrence on the same template patient. This approach provided a clearer and more explicit depiction of recurrence patterns, making the locations immediately apparent and offering a more precise basis for defining target areas in adjuvant radiotherapy.

Our results showed that 59.3% (35/59) of recurrence sites occurred within proximity of the SMA (mean distance: 2.56 cm; range: 0.66-8.57 cm), while 40.7% (24/59) clustered near the CA (mean distance: 2.07 cm; range: 0.67-6.79 cm). Patients with PDTC were more likely to experience recurrence near the SMA after surgery. Moreover, multiple high-quality studies have consistently reported that postoperative local recurrence in pancreatic cancer predominantly involves the perivascular regions of the SMA and CA, with SMA-proximal lesions constituting 69%-77% of all documented local recurrences (10–12). Optimally, this spatial predilection may reflect the propensity of pancreatic cancer to directly infiltrate the SMA adventitia and impair venous drainage, thereby fostering a microenvironment conducive to local tumor regrowth (14). Regarding therapeutic management, adjuvant radiotherapy might alter the recurrence pattern post-surgery (15). Consequently, patients who received adjuvant radiotherapy after surgery were excluded from this study to enhance the reliability of the findings. Although adjuvant chemotherapy improved patient survival prognosis, its effect on altering the recurrence pattern following PDTC surgery remained unclear (5). In this study, patients were further stratified based on whether they received chemotherapy. Among the patients who received adjuvant chemotherapy, 40.7% (24/59) experienced local recurrence—of which 54.2% (13/24) were located near the CA and 37.5% (9/24) near the SMA. In contrast, among the patients who did not receive adjuvant chemotherapy, 59.3% (35/59) developed local recurrence—of which 68.6% (24/35) occurred near the SMA and 31.4% (11/35) near the CA. The studies indicated that adjuvant chemotherapy following surgical resection may reduce the risk of local recurrence (16). In line with this evidence, patients who received adjuvant chemotherapy in the present cohort exhibited a numerically lower local recurrence rate compared with those who did not. However, this difference did not reach statistical significance—likely attributable to limited statistical power arising from the relatively small sample size. Based on whether patients received adjuvant chemotherapy, there was a tendency in the target area for adjuvant radiotherapy. For patients who received adjuvant chemotherapy, the target area for adjuvant radiotherapy should focus more on the region near the CA, while for those who did not receive adjuvant chemotherapy, the target area should focus more on the region near the SMA. This difference might be attributed to the inconsistent concentration of chemotherapy drugs in tissues surrounding the CA and SMA (17, 18). Additionally, it could also be related to changes in gene characteristics induced by adjuvant treatment, which might alter metastasis pathways (19).

Previous studies have attempted to map local recurrence patterns to evaluate the rationality of the target volume for adjuvant radiotherapy after surgery for intrahepatic cholangiocarcinoma. The results indicated that postoperative recurrence in distal extrahepatic cholangiocarcinoma predominantly occurred at the biliary-enteric anastomosis, which could not be effectively covered by CTV_RTOG_. This finding was valuable for designing the delineation range of the target volume (20). Currently, there are limited studies on target volume delineation for adjuvant radiotherapy after surgery for PDTC, with most references relying on the RTOG 0848 consensus guidelines for postoperative adjuvant radiotherapy targeting pancreatic head cancer, excluding the hepatic portal area (9). This study primarily included patients with pancreatic body and tail cancer after surgery and investigated whether the current RTOG consensus guidelines for pancreatic cancer target volumes adequately cover recurrent lesions. However, when applying the RTOG target volume, approximately 40.68% of recurrence points were not covered by the postoperative adjuvant radiotherapy target volume, including about 62.5% of recurrence points near the CA and about 25.71% near the SMA. Thus, the adjuvant radiotherapy target volume inadequately covered recurrent lesions, particularly near the CA, leading to suboptimal local recurrence control. Conversely, the current study showed that more recurrence points occur near the SMA, but when the RTOG consensus guidelines for pancreatic head cancer are applied to adjuvant radiotherapy after surgery for PDTC, the local recurrence sites near the SMA exhibited sufficient coverage. Our prior research indicated that patients with PDTC might derive little benefit from adjuvant radiotherapy, which we hypothesize could be related to the delineation of target areas in postoperative adjuvant radiotherapy (8). Based on these findings, patients were stratified according to whether they received postoperative adjuvant chemotherapy. Among patients who received chemotherapy, 50.00% (12/24) of recurrence points were not covered, compared to 34.29% (12/35) in patients who did not receive chemotherapy. Therefore, the target volume for postoperative adjuvant radiotherapy in patients who received chemotherapy requires further adjustment, potentially due to differences in postoperative recurrence patterns among these patients (21). For solid tumors undergoing chemotherapy, drugs delivered via the bloodstream must cross the vascular wall and surrounding tissues to reach tumor cells at varying distances from the nearest blood vessels. These transport processes depend not only on drug properties but also on physiological characteristics, including the structure and flow distribution of the microvascular system supplying the tumor, as well as the nature of extracellular tissue components such as the extracellular matrix (ECM), normal cells, tumor cells, and interstitial spaces. In patients receiving chemotherapy, drug concentrations are higher near blood vessels, inhibiting tumor cell proliferation in these regions, whereas lower drug concentrations at distant sites allow easier proliferation. Recurrence lesions outside the conventional adjuvant radiotherapy target volume range are therefore difficult to control (22). In the chemotherapy group, 9 of 13 (69.2%) recurrent lesions near the CA were outside the planned target volume, compared with only 3 of 11 (27.3%) near the SMA. This suggests that the current CTV margin around the CA warrants expansion in patients receiving adjuvant chemotherapy. In contrast, among patients who did not receive chemotherapy, although SMA-proximal recurrences predominated (24/35, 68.6%), a substantial proportion—6 of 24 (25.0%)—remained uncovered; notably, among the 11 CA-proximal recurrences (31.4% of all recurrences), 6 (54.5%) were also outside the CTV. Thus, suboptimal coverage of CA-adjacent disease was observed irrespective of chemotherapy status. The median distances from recurrence sites to the SMA and CA were 2.56 cm and 2.07 cm, respectively. According to our RTOG-compliant contouring protocol, the CTV margin is defined as 1.0 cm proximal to the origin of the CA and 3.0 cm proximal to the origin of the SMA. Given that the observed recurrence distances exceed these margins—particularly for the CA—the current CA margin appears insufficient, supporting a rationale for systematic expansion of the CA-related CTV margin.

Our analysis revealed that 46.3% of local recurrences were concurrent with distant metastasis, with hepatic involvement being the most frequent (34.2%), followed by pulmonary (12.29%), pleural (12.29%), and osseous (4.88%) metastases. In contrast, Honselmann et al. reported that among 546 patients undergoing resection for PDAC between 2005 and 2016, distant metastasis occurred in 62%, isolated local recurrence in 24%, and combined local–distant recurrence in 14%; hepatic metastases accounted for 39% of all distant failures, with pulmonary metastases ranking second (23). Notably, that cohort included patients with pancreatic head cancer—whereas tumors of the pancreatic body and/or tail are associated with a higher propensity for systemic dissemination (24). This anatomical distinction likely contributes to the observed divergence in recurrence patterns: in our cohort—restricted to patients with locally recurrent disease—nearly half (46.3%) of local recurrences co-occurred with distant metastasis, a proportion substantially higher than the 14% combined pattern reported by Honselmann et al. Furthermore, although adjuvant chemotherapy demonstrated a trend toward improved OS and DFS in patients with PDTC, no statistically significant benefit was observed—unlike findings from prior studies (8). This discrepancy may reflect selection bias inherent in our study design, as enrollment was limited exclusively to patients with documented local recurrence and excluded those presenting with *de novo* distant metastasis alone. Perineural invasion (PNI) is a frequently encountered pathological feature in pancreatic ductal adenocarcinoma and has been well established as not only an independent prognostic factor for DFS (25), but also a significant predictor of OS (26–28). A multicenter retrospective study reported a PNI positivity rate of 87% among surgically resected pancreatic ductal adenocarcinoma specimens, with variation across tumor stages (61.6% in T0–1; 86.7% in T2; 90.1% in T3–4; 72.5% in N0; and 93.0% in N1) (29). In the current cohort of patients with PDTC, perineural invasion was identified in 18 out of 41 cases (43.90%), a rate notably lower than those reported in the existing literature. This discrepancy may be attributed to selection bias arising from the study’s inclusion criteria, specifically the restriction to patients with locally recurrent disease and availability of high-quality imaging follow-up, which may limit the generalizability of the findings and affect the representativeness of the sample.

This study has several limitations. First, current clinical guidelines recommend postoperative adjuvant radiotherapy for patients with pathologically confirmed nodal involvement or positive resection margins following pancreatic cancer surgery. However, due to the limited sample size, our cohort lacked sufficient power to stratify analyses by these established high-risk features. Instead, stratification was performed solely according to receipt of adjuvant chemotherapy. Given emerging evidence that distinct chemotherapy regimens—such as gemcitabine-based versus FOLFIRINOX—may differentially modulate local tumor control and recurrence biology, the absence of regimen-specific subgroup analyses limits the robustness and generalizability of our comparisons. Second, recurrence localization relied exclusively on serial cross-sectional imaging (CT/MRI) without histopathological confirmation via biopsy. Although we meticulously mapped recurrence sites to correspond as closely as possible to their anatomically verified locations on diagnostic imaging, inherent biological uncertainties—including microscopic spread, treatment-induced morphologic changes, and inter-observer variability in image interpretation—may introduce non-negligible spatial discrepancies between assigned and true recurrence coordinates. Third, as a single-institution retrospective analysis, this study was designed to characterize recurrence patterns and assess existing target volume coverage—not to prospectively define or validate novel CTV delineation strategies. Consequently, while our findings suggest potential refinements to CTV-related margins, the optimal geometric expansion remains undefined and warrants validation in prospective, multicenter trials incorporating standardized imaging protocols and central radiologic review.

## 5 Conclusion

In conclusion, we developed a 3-D anatomical recurrence map for patients with PDTC following radical resection and quantitatively evaluated the coverage of local recurrence lesions by the CTV_RTOG_, revealing that 40.68% of recurrence sites fell outside this target volume. These findings may provide valuable insights into the construction of the target volume for postoperative radiotherapy in PDTC.

## Data Availability

The original contributions presented in the study are included in the article/supplementary material. Further inquiries can be directed to the corresponding author.
